# Epigenetic Perspectives on Maternal Gut Microbiota's Impact on Embryonic and Fetal Development

**DOI:** 10.1002/cph4.70163

**Published:** 2026-05-05

**Authors:** Shoulong Xu, Yanjiao Huang, Chenyue Hu, Wenli Ding, Zhenwei Pu, Qiang Li, Zhiliang Xu

**Affiliations:** ^1^ Human Anatomy Experimental Training Center, School of Basic Medical Science Wannan Medical University Wuhu Anhui China; ^2^ DD&E Institute, School of Basic Medical Sciences Wannan Medical University Wuhu Anhui China; ^3^ School of Clinical Medicine Wannan Medical University Wuhu Anhui China; ^4^ Institutes of Brain Science Wannan Medical University Wuhu Anhui China; ^5^ School of Pharmacy Wannan Medical University Wuhu Anhui China; ^6^ Department of Human Anatomy, School of Basic Medical Science Wannan Medical University Wuhu Anhui China; ^7^ Anhui Province Key Laboratory of Basic Research and Translation of Aging‐Related Diseases Wannan Medical University Wuhu Anhui China

**Keywords:** embryonic and fetal development, epigenetics, maternal gut microbiota, maternal‐fetal interaction, nutritional intervention

## Abstract

Exposure to harmful environments during pregnancy and maternal nutritional status are key factors that affect offspring development; however, the underlying mechanisms of maternal–fetal interaction remain to be elucidated. In recent years, research on gut microbiota and epigenetics has provided new perspectives for understanding these mechanisms. This review systematically summarizes the potential mechanisms by which the maternal gut microbiota influences prenatal development from an epigenetic perspective. Furthermore, it discusses the role of personalized nutritional interventions in the prevention of non‐communicable diseases during embryonic and fetal development, aiming to provide new insights and intervention targets for promoting healthy pregnancies and enabling early disease prevention.

## Graphical abstract

This review summarizes how maternal health and nutrition shape gut microbiota via epigenetics to regulate embryonic development. It highlights microbiota‐embryo interactions, disruption by prenatal chemical exposures, and personalized nutrition for disease prevention, offering novel insights and therapeutic targets.

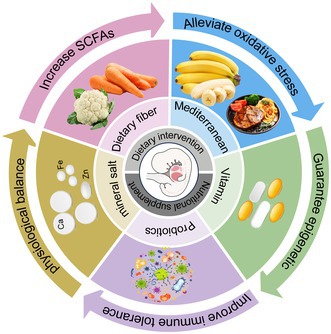

## 1 Introduction

During fetal formation, the embryonic period (from the 1st week to the 8th week post‐fertilization) encompasses three key developmental milestones: establishment of the three germ layers (endoderm, mesoderm, and ectoderm), formation of the neural tube (precursor to the brain and spinal cord), and emergence of vital organs like the heart, lungs, liver, and kidneys. This critical phase not only determines the structure of major organs but also represents the most vulnerable window for teratogenic factors (Rossant and Tam 2022; Qi et al. 2025). At this stage, although the embryonic stage laid the foundation of organ morphology, the influence of environmental factors can often continue to the fetal stage. From the ninth week to the end of delivery, it entered the fetal period. This stage is the key period of human body shaping after the embryonic period, which is mainly manifested in the further maturity of various organs, a perfect body structure, and rapid growth.

Recent maternal‐fetal interaction studies have demonstrated that maternal gut microbiota plays a crucial role during pregnancy (Ziętek et al. 2021). Placenta is an important organ connecting the fetus and the mother, which is mainly responsible for material exchange, endocrine regulation and immune regulation during pregnancy to maintain pregnancy and ensure the normal development of the fetus (Cindrova‐Davies and Sferruzzi‐Perri 2022). In a healthy physiological state, the role of the intestinal barrier and placental barrier can selectively regulate the transfer of molecules, allow necessary nutrients and immune factors to pass through, and limit the entry of harmful substances such as LPS, thus maintaining the stability of the uterine environment (Suzuki 2020; Tetro et al. 2018). In this process, gut microbiota performs vital biological synthesis and regulatory functions: it reprocesses food to synthesize essential micronutrients and ferment dietary fiber to produce short‐chain fatty acids (SCFAs). These actions help maintain maternal immune balance, suppress harmful inflammatory responses, and provide a stable developmental environment for the fetus (Cao et al. 2025; Gou et al. 2025; Wang, Chai, et al. 2024; Huang et al. 2025).

Epigenetics provides an ideal perspective for exploring maternal gut microbiota's influence on embryonic development. Since David Barker proposed the “Developmental Origins of Health and Disease” (DOHaD) theory in 1995, researchers have increasingly recognized the crucial role of epigenetic mechanisms in embryonic programming and disease onset (Lapehn and Paquette 2022). The placenta, serving as a vital bridge between mother and fetus, plays a central role in regulating embryonic nutrient supply and developmental microenvironment. It transmits biological information from maternal gut microbiota changes to the embryo through uterine spiral arteries and umbilical veins, establishing a dynamic “mother‐placenta‐fetus” regulatory axis that continuously influences embryonic development (Cindrova‐Davies and Sferruzzi‐Perri 2022). When maternal gut microbiota becomes dysregulated, impaired intestinal barrier function may lead to deficiencies in key metabolites and nutrients such as short‐chain fatty acids and folic acid (Chi et al. 2017; Zhang, Liu, et al. 2025). These changes may further disrupt normal epigenetic modifications in the embryo, including histone modifications and DNA methylation, thereby increasing the risk of fetal brain abnormalities and structural malformations (Socha et al. 2024; Gurugubelli and Ballambattu 2024). Maternal gut microbiota's regulatory mechanisms also correlate with maternal nutritional status and environmental exposures. For instance, maternal beta‐carotene supplementation may improve embryo growth and developmental defects by enhancing intestinal immune function and modulating maternal gut microbiota composition (Wang, Wang, et al. 2025). The Mediterranean diet can increase the abundance of Ruminococcaceae, Acidaminococcaceae, and Bacteroidaceae in the intestines of postpartum newborns and change the methylation levels of NCK2, SNED1, MTERF4, MSH5, and HLA‐DPB1 genes (Sasaki et al. 2023). These findings suggest that the changes of maternal nutritional status and intestinal microbial composition may indirectly affect the development of embryos and fetuses through epigenetic modification. Further investigation into the epigenetic impacts of maternal gut microbiota on embryonic and fetal development will deepen our understanding of developmental defects and provide scientific foundations for preventive strategies. This review systematically summarizes existing research to elucidate how maternal gut microbiota influences embryonic development through epigenetic regulatory mechanisms, while exploring the potential applications of personalized nutrition in this process.

## 2 Maternal Adaptations in Pregnancy

The placenta serves as a vital organ for embryos and fetuses to sense maternal changes, responsible for gas exchange and nutrient supply during their development. Its barrier function can selectively block most bacteria and pathogens, safeguarding the embryonic microenvironment. Notably, maternal gut microbiota can indirectly influence embryonic and fetal development through short‐chain fatty acids and immune factors secreted by the mother. Furthermore, changes in maternal gut microbiota are closely associated with various pregnancy complications such as preeclampsia, gestational diabetes, intrahepatic cholestasis of pregnancy, and preterm birth (Table 1) (Xiong et al. 2023; Liu et al. 2017; Sun et al. 2020; Liu, Chen, et al. 2024; Su et al. 2021; Liu, Sun, et al. 2023; Balci et al. 2022). These complications significantly determine pregnancy outcomes. Therefore, studying maternal gut microbiota and its metabolites' roles in establishing the embryonic and fetal microenvironment and regulating the immune system is crucial for understanding the intrinsic mechanisms of maternal‐fetal interactions.

**TABLE 1:** Changes in maternal gut microbiota during the occurrence of pregnancy complications.

Complications of pregnancy	Changes in maternal gut microbiota	Species	References
Preeclampsia	Streptococcus ↓ Acinetobacter ↓ C. diffucens↑ Bradyella↑ Bifidobacterium ↓ Lactobacillus ↓	Human Human Wistar rat	(Xiong et al. 2023) (Liu et al. 2017) (Sun et al. 2020)
Hepatic cholestasis of pregnancy	Bacteroidetes ↑ Bacillus ↑ Sphingolobus ↑ Blautia ↑ Lactobacillus ↑	Human	(Liu, Chen, et al. 2024)
Gestational diabetes	Deinococcus ↓ Bacteroidetes ↑ Bifidobacterium ↓ Blautia ↑ Nodularia ↑ Bacteroides ↑	Human Human	(Su et al. 2021) (Liu, Sun, et al. 2023)
Hyperemesis gravidarum	Clostridium ↑ Candida ↑ Bifidobacterium ↓	Human	(Balci et al. 2022)

### 2.1 Composition and Dynamic Changes of Maternal Gut Microbiota

**FIGURE 1:**
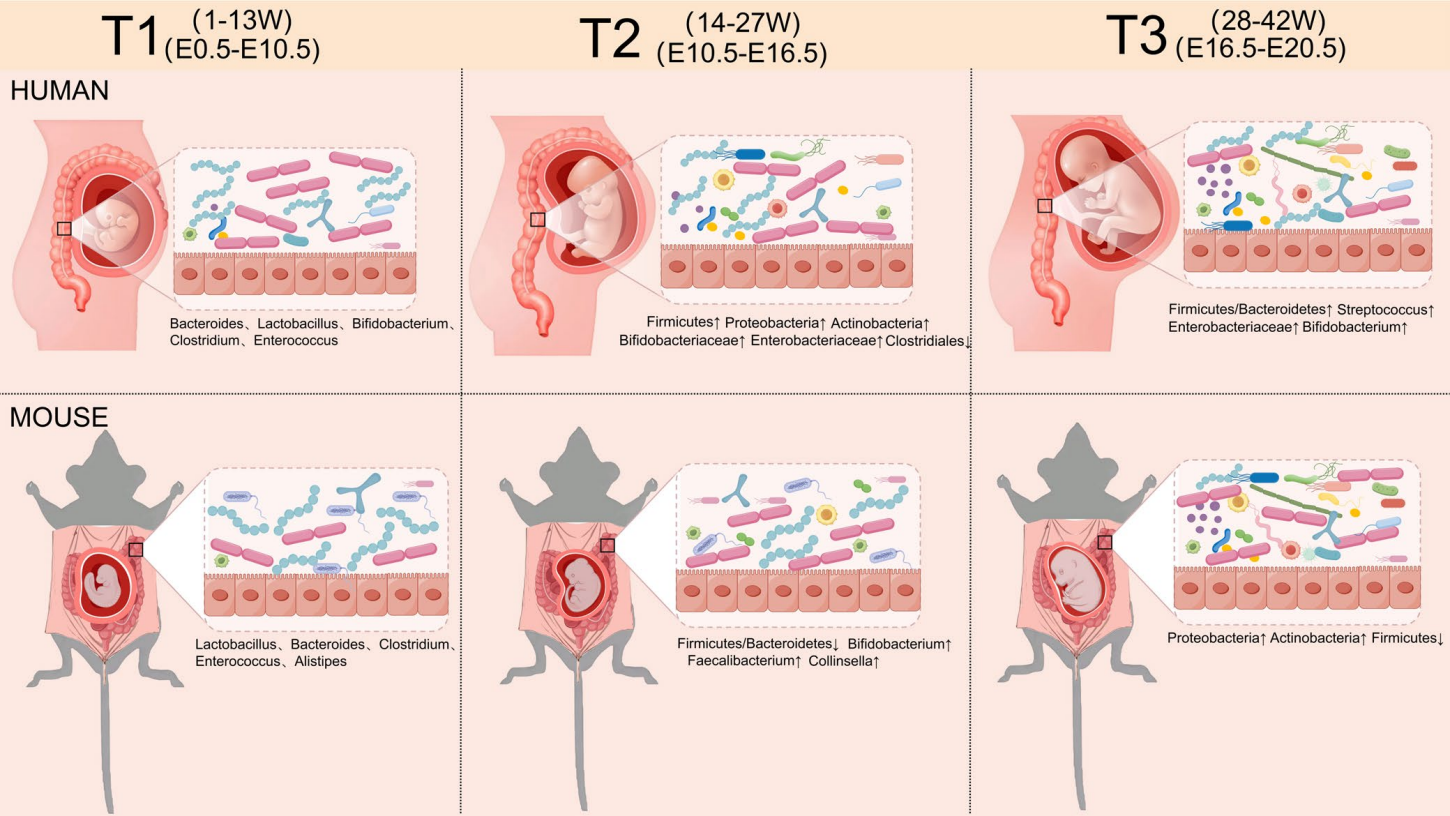
Schematic illustration of dominant intestinal bacterial genera in humans and mice embryonic development. Maternal gut microbiota changes during early pregnancy (T1), mid‐pregnancy (T2), and late pregnancy (T3).

As the “second genome” of the human body, most microorganisms reside in the gut. In normal non‐pregnant women, the gut microbiota is predominantly composed of Firmicutes and Bacteroidetes, accounting for 90% of the microbial community, followed by Actinobacteria, Proteobacteria, Fusobacteria, and Cystobaculales, which facilitate food digestion, nutrient synthesis, immune regulation, and barrier maintenance (Yan et al. 2023; Bhatia et al. 2024). At the phylum level, non‐pregnant mice share high similarity with humans, primarily consisting of Firmicutes and Bacteroidetes, accompanied by minor proportions of Proteobacteria and Actinobacteria. Significant genus‐level differences exist between mice and humans: non‐pregnant mice predominantly harbor Lactobacillus and Bacteroides genera, while humans mainly possess Bacteroides and Prevotella genera (Wang et al. 2022; Ghosh and Pramanik 2021). Maternal gut microbiota undergoes notable changes during pregnancy, directly impacting maternal metabolism and immune status (Yan et al. 2023) (Figure 1). In early pregnancy (T1), the gut microbiota structure resembles that of non‐pregnant women, dominated by Firmicutes and Bacteroidetes, with minor variations including Bifidobacterium and Klebsiella genera, laying the foundation for subsequent metabolic changes, energy supply, and immune regulation (Bhatia et al. 2024; Tian et al. 2024). During early pregnancy in mice, the abundance of Proteobacteria decreases while Bacteroides and Akkermansia genera begin to enrich (López‐Agudelo et al. 2024). In mid‐pregnancy (T2), alpha diversity gradually declines while beta diversity increases, specifically manifested as rising abundance of Firmicutes, Proteobacteria, Actinobacteria, Bifidobacteriaceae, and Enterobacteriaceae genera, alongside decreasing abundance of Cystobaculales (Yan et al. 2023; Bhatia et al. 2024; Tian et al. 2024). During mid‐pregnancy in mice, the ratio of Firmicutes to Bacteroidetes decreases, while Bifidobacterium shows significant enrichment, with slight increases in Enterobacteriaceae and Collinsella (López‐Agudelo et al. 2024). In late pregnancy (T3), alpha diversity reaches its lowest point, accompanied by increased abundance of Firmicutes (e.g., Streptococcus), Proteobacteria (e.g., Enterobacteriaceae), and Actinobacteria (e.g., Bifidobacterium), supporting the fetal growth sprint and preparing for delivery (Bhatia et al. 2024; Tian et al. 2024; Koren et al. 2024). In late pregnancy, Proteobacteria and Actinobacteria show significant increases, Firmicutes abundance continues to decline, and Bifidobacterium peaks (López‐Agudelo et al. 2024). Maternal gut microbiota composition during pregnancy adapts to factors like maternal health status, hormonal changes, and diet, ultimately reaching equilibrium (Zhang, Liu, et al. 2025; Amato et al. 2024).

### 2.2 Maternal Systemic Adaptations

The embryonic microenvironment refers to the localized and dynamic extracellular environment surrounding the embryo before and after implantation. It consists of maternal‐derived nutrients and hormones, a complex and dynamic extracellular matrix, and intercellular signaling networks, forming a three‐dimensional spatial structure. This environment interacts with embryonic cells to collaboratively regulate embryonic growth, differentiation, and tissue formation. The following discussion focuses on the changes in maternal metabolism, endocrine function, and immunity during pregnancy and their impact on embryonic and fetal development.

#### 2.2.1 Maternal Metabolic and Endocrine Adaptations

In early pregnancy, in order to ensure the supply of nutrition and the successful implantation of embryos during pregnancy, maternal metabolism showed a tendency of normal insulin sensitivity and decreased fasting blood sugar. At the same time, the metabolism of amino acids such as alanine, aspartic acid and glutamic acid is significantly enhanced, which provides energy and material basis for uterine decidualization and embryo implantation (Lin et al. 2024). In addition, the level of human chorionic gonadotropin (hCG) in pregnant women at this stage increases, which stimulates the corpus luteum to produce progesterone and estrogen, and participates in angiogenesis and placental development (Enache et al. 2025). In the second trimester, the increase of human placental prolactin (hPL) secretion began to drive pregnant women's physiology to catabolism, mainly manifested in the progressive decline of insulin sensitivity (Enache et al. 2025). In addition, in order to meet the nutritional needs of the rapid growth of the fetus, the maternal blood volume expands and the lipid metabolism pathway is activated, which leads to the occurrence of physiological hyperlipidemia (Blanco Sequeiros et al. 2023). In the third trimester, hPL increased further and insulin resistance reached its peak. At the same time, the fetal signal molecule DLK1 secretes into maternal circulation, regulates maternal liver lipogenesis and skeletal muscle fatty acid oxidation, enhances fat mobilization, and significantly increases free fatty acids and triglycerides (Kosmas et al. 2025; Cleaton et al. 2016).

#### 2.2.2 Maternal Immune Adaptations

During pregnancy, the maternal immune system maintains a dynamic balance, thus ensuring the establishment of immune tolerance to embryos and resisting the invasion of foreign pathogens. In early pregnancy, pregnant women are in a state of promoting inflammation, which is conducive to embryo implantation. When the embryo is implanted into the endometrium, it causes a local inflammatory reaction, and a large number of immune cells are recruited to the implantation site to remove cell debris and reshape blood vessels, creating conditions for the formation of placenta (Joo et al. 2024). In the second trimester, the maternal immune system changes its anti‐inflammatory mode, thus establishing immune tolerance against embryos. At this stage, the number of regulatory T cells (Tregs) increased significantly. By secreting IL‐10, TGF‐β and other anti‐inflammatory factors, the activation of effector T cells was inhibited to prevent them from attacking the fetus. At the same time, decidual natural killer cells (dNK) secrete angiogenic factors to promote uterine spiral artery remodeling and ensure adequate blood flow to the placenta (Joo et al. 2024; Liu, Liang, et al. 2025). In addition, the expression level of Toll‐like receptor 9 (sTLR9) on the surface of neutrophils in maternal peripheral blood is increased, which is also proved to be helpful to maintain the immune hyporesponsiveness (Wang, Lu, et al. 2025). In the third trimester, it turns to a pro‐inflammatory state again to prepare for delivery. Inflammatory cell infiltration occurs locally, releasing inflammatory mediators such as IL‐1β, IL‐6 and IL‐8, triggering cervical maturation and uterine contraction (Liu, Liang, et al. 2025).

### 2.3 Placental Transport of SCFAs and Its Regulation on Embryo and Fetal Development

The primary metabolic products of gut microbiota include SCFAs (such as acetic, propionic, and butyric acids), bile acids and their derivatives (including primary and secondary bile acids), amino acid‐related metabolites (such as branched‐chain amino acids, tryptophan metabolites like indole and its derivatives, histidine metabolites like imidazolpropionic acid), as well as other physiologically active molecules including lipopolysaccharides (LPS) and trimethylamine (TMA) (Zhang, Jian, et al. 2023; Su et al. 2022; Tao and Ma 2024). SCFAs are organic fatty acids composed of 1–6 carbon atoms. In the human body, acetic, propionic, and butyric acids dominate, accounting for over 90% of total SCFAs (Mansuy‐Aubert and Ravussin 2023). Different bacterial species produce distinct SCFAs through specific metabolic pathways (Table 2) (Zhang, Jian, et al. 2023; Hays et al. 2024; Fusco et al. 2023; Makki et al. 2018): Acetic acid is synthesized by anaerobic bacteria such as Adibacterium adiinum, Bacteroides, and Bifidobacterium via acetyl‐CoA conversion; propionic acid is primarily produced by Bacteroides and Prevotella through the succinate pathway; butyric acid is synthesized by Clostridium clusters IV and XIVa, as well as Praesens, through the condensation of two acetyl‐CoA molecules into butyryl‐CoA, followed by synthesis via the butyrate kinase pathway or butyryl‐CoA:acetyl‐CoA transferase pathway (Zhang, Jian, et al. 2023; Koh et al. 2016).

**TABLE 2:** Short‐chain fatty acid biosynthetic pathways and gut microbiota involvement.

SCFAs	Action pathways	Bacterial gut microbiota involvement	Document
Acetic acid	Acetyl‐CoA pathway	*Akkermansia muciniphila* , Bacteroides spp., Bifidobacterium spp., Prevotella spp., Streptococcus spp., *Blautia hydrogenotrophica* , Clostridium spp., Ruminococcus spp	(Zhang, Jian, et al. 2023) (Hays et al. 2024) (Fusco et al. 2023)
	Wood‐Ljungdahl pathway	Acetogenic bacteria, Clostridium spp., Streptococcus spp., *Blautia hydrogenotrophica*	(Zhang, Jian, et al. 2023) (Hays et al. 2024) (Makki et al. 2018)
Propionic acid	Succinate pathway	Bacteroides spp., Prevotella spp., Bacteroidetes phylum, Negativicutes class, *Dialister succinatiphilus* , Phascolarctobacterium, succinatutens, Veillonella spp., Dialister spp	(Zhang, Jian, et al. 2023) (Hays et al. 2024; Fusco et al. 2023; Makki et al. 2018)
	Acrylic acid pathway	*Coprococcus catus* , Megasphaera spp., Coprococcus spp., *Megasphaera elsdenii*	(Zhang, Jian, et al. 2023) (Hays et al. 2024; Fusco et al. 2023; Makki et al. 2018)
	Propylene glycol pathway	*Akkermansia muciniphila* , Blautia wexleri, *Roseburia inulinivorans* , *Ruminococcus obeum* , *Salmonella enterica* serovar Typhimurium, Blautia, Salmonella spp	(Zhang, Jian, et al. 2023) (Hays et al. 2024; Fusco et al. 2023; Makki et al. 2018)
Butyrate	Butyrate kinase pathway	Coprococcus spp., *Coprococcus comes* , *Coprococcus eutactus*	(Zhang, Jian, et al. 2023) (Hays et al. 2024) (Makki et al. 2018)
	D‐toluidyl‐CoA: Acetyl‐CoA transferase pathway	Anaerostipes spp., *Clostridium leptum* , *Coprococcus catus* , *Eubacterium rectale* , Eubacterium halli, *Faecalibacterium prausnitzii* , Roseburia spp., *Ruminococcus bromii*	(Zhang, Jian, et al. 2023) (Hays et al. 2024) (Makki et al. 2018)

#### 2.3.1 Placental Transport Mechanisms of SCFAs

In the early stage of placenta formation, SCFAs can stimulate vascular endothelial cells in placenta to form new vascular branches, and prevent placental growth restriction and vascular insufficiency (Pronovost et al. 2023). At the same time, SCFAs can also activate PI3K‐Akt signaling pathway in placenta to alleviate oxidative stress and inflammatory reaction in placenta (Feng, Wu, et al. 2025). These compounds activate G protein‐coupled receptors (GPCRs) in placental tissues (e.g., GPR41, GPR43, and GRP109A) and are subsequently converted into acetyl‐CoA. This metabolic process regulates fetal signal receptor activation, histone acetylation, and energy metabolism, thereby influencing placental function and embryonic development (Zhang, Jian, et al. 2023; Su et al. 2022; Xu, Zhou, et al. 2022; Sun et al. 2021; Qin et al. 2025).

#### 2.3.2 Regulatory Mechanisms of SCFAs in Embryonic and Fetal Development

**FIGURE 2:**
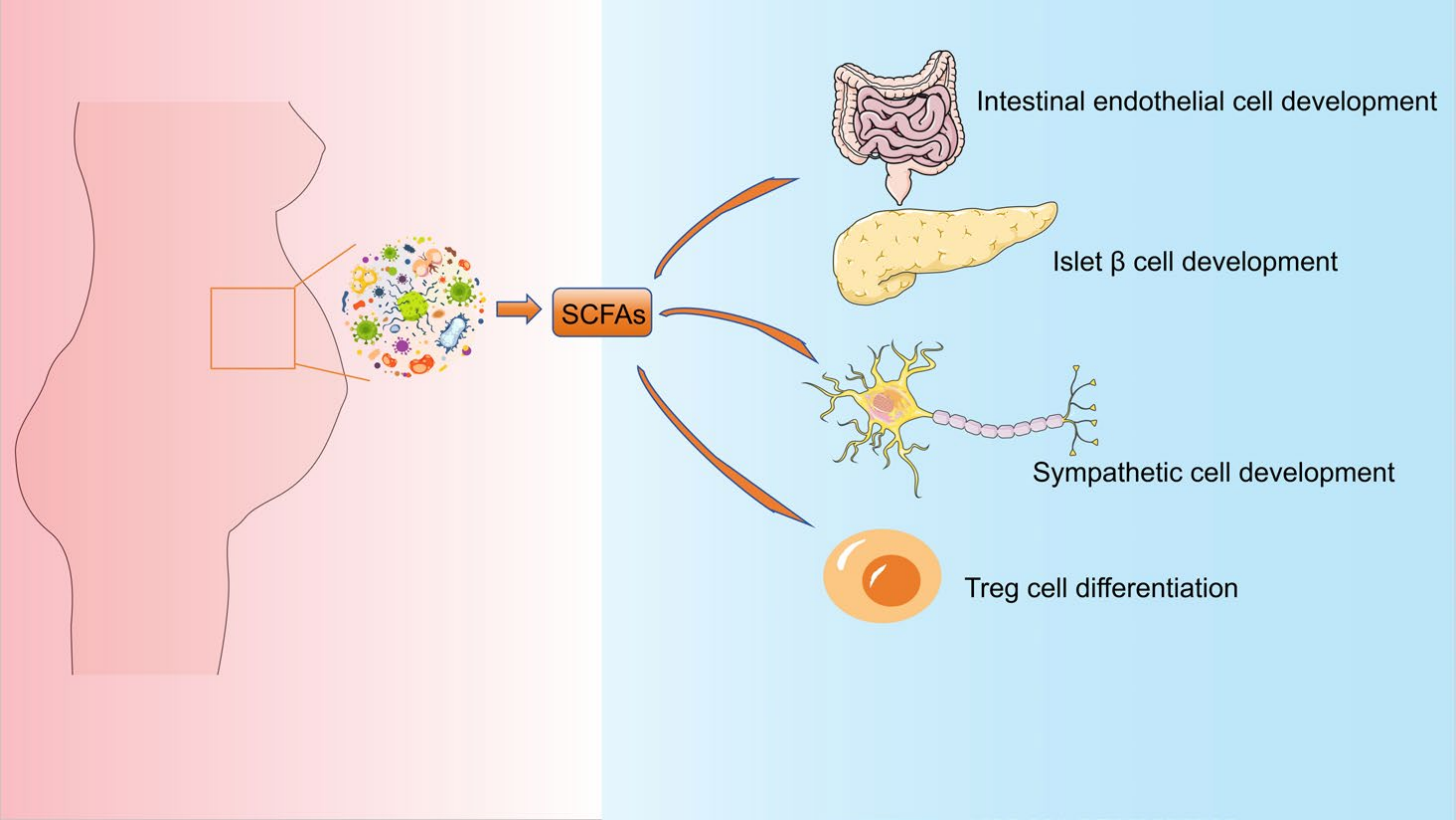
SCFAs influence embryonic development across multiple systems. These SCFAs, produced by maternal gut microbiota, directly regulate the development of the embryo's metabolic, nervous, and immune systems through the placental barrier.

Maternal‐derived SCFAs exert profound effects on embryonic and fetal organ development (Figure 2). Research by Ikuo Kimura's team demonstrates that propionates enhance the development of glucagon‐like peptide‐1 (GLP‐1) expressing intestinal endocrine cells and pancreatic β‐cells by activating GPR43 in the embryo's gut and pancreas, ensuring stable plasma glucose and insulin levels (Kimura et al. 2020). As the most potent agonist of GPR41, propionates also promote the development of embryonic sympathetic nerve cells. In GPR41 gene knockout embryos and germ‐free maternal mice, cardiac sympathetic nerve projections were significantly reduced, a defect that was improved by propionate supplementation (Kimura et al. 2020). In addition, propionic acid can also activate the cGMP‐PKG signaling pathway by inducing lactate of histone H4K12 in the fetal brain, up‐regulate the transcription factor Sox family necessary for oligodendrocyte differentiation, and regulate fetal myelination (Zhang et al. 2026). Maternal SCFAs further support the establishment of the fetal immune system, reducing the risk of immune‐related disorders such as allergies, asthma, and autoimmune diseases in the postpartum period (Mukhopadhya and Louis 2025; Vuillermin et al. 2017). Research by Mingjing Hu's team demonstrates that propionic acid and butyric acid may promote the differentiation of regulatory T cells (Tregs) by inhibiting histone deacetylases (HDACs), thereby regulating histone H4 acetylation modifications (K5ac, K8ac, K12ac, and K16ac) and facilitating the establishment of fetal immune tolerance mechanisms (Hu et al. 2022). Future interventions targeting the maternal gut microbiota‐short‐chain fatty acids‐fetal development axis could provide novel therapeutic approaches for preventing metabolic diseases, immune disorders, and neurodevelopmental abnormalities.

### 2.4 The Association Between Maternal Gut Microbiota Imbalance and Prenatal Dysplasia

There is still controversy about whether there are microorganisms in the uterus. At present, the mainstream thinking tends to suggest that the microorganisms in the mother's intestines do not directly enter the uterus to affect the fetus, but through the “gut‐reproductive axis” to integrate environmental signals, affect the placental function and the metabolism, immunity and endocrine status of the mother, and indirectly affect the development of the fetus (Moustakli et al. 2025).

#### 2.4.1 Signaling Mechanisms of Maternal Gut Microbiota in the Placenta

In early pregnancy, the loss of maternal gut microbiota can affect placental vascular density and maturity by increasing the levels of angiogenic proteins (VEGF‐A, FGF‐2) and inhibiting downstream signals (p‐ERK1/2) (Coskun et al. 2025). Thiamine from gut microbiota can activate the placental Notch signaling pathway and then start the PI3K/AKT signaling cascade and promote placental angiogenesis and nutrient transport efficiency (Sun et al. 2025). Exosomes from the placenta, as extracellular vesicles, can deliver signal molecules regulated by gut microbiota to placental trophoblasts and regulate the immune microenvironment and endothelial function at the maternal‐fetal interface (Ma, Cao, et al. 2025). Tryptophan metabolites produced by gut microbiota promote the recruitment of myelogenous inhibitory cells (MDSCs) and RORγt+ regulatory T cells (pTregs) to the placenta and maintain the maternal tolerance to the fetus (Brown et al. 2026). When the imbalance of gut microbiota causes abnormal tryptophan metabolism, excessive kynurenic acid and xanthuric acid will be produced. These harmful metabolites cross the placental barrier and inhibit the IFN‐β signaling pathway in the placenta and fetal brain, resulting in impaired development of the fetal blood–brain barrier (Wang et al. 2026). In addition, the imbalance of gut microbiota can also affect the IFN‐γ secretion of NK cells by regulating placental carbohydrate metabolism (Giugliano et al. 2025).

#### 2.4.2 Impact of Preconception Maternal Gut Dysbiosis on Oocyte Development

The imbalance of intestinal microorganisms is related to polycystic ovary syndrome (PCOS), endometriosis, infertility, and pregnancy complications (Moustakli et al. 2025). Taking obesity as an example, studies show that the maternal gut microbiota imbalance of obese people is closely related to the quality of oocytes (Li et al. 2023; Orvieto et al. 2020). The transplantation of obesity‐related microbiota and the decrease of Christensenellacea r‐7 group's abundance induced by a high‐fat diet can lead to abnormal meiosis of oocytes, damage to maternal mRNA, decline in egg quality, and impairment of embryo development ability by damaging pyrimidine metabolism of ovaries, reducing butyric acid level, and water equality of 5mC and H3K36me3 in ovarian tissues (Zeng et al. 2023; Shan et al. 2024; Qi et al. 2024), and obesity can also induce female infertility and pregnancy complications. For example, the decrease of Stella protein in the oocytes of obese mice will cause hypomethylation of the whole genome and lead to embryonic development defects (Han et al. 2018).

#### 2.4.3 Regulation of Embryonic Development by Gut Microbiota

The imbalance of maternal gut microbiota is significantly associated with abnormal embryonic development, particularly neural system development. This imbalance may lead to increased inflammatory responses, thereby affecting embryonic cell differentiation and organ formation (Vuong et al. 2020; Minakova and Warner 2018; Yan, Shi, et al. 2025; Yao et al. 2025). The imbalance of maternal gut microbiota will increase the level of LPS and hinder fetal neurogenesis by activating NF‐κB and IL‐6 signals and destroy the normal closure of embryonic neural tubes. Interestingly, inflammation‐related embryonic development disorders can be alleviated by the intake of probiotics. When the microbiota of normal mice is transplanted, the level of LPS in serum can be reduced and the neurogenesis inhibition caused by microbiota imbalance can be significantly reversed (Long et al. 2021). Probiotics *Lactobacillus acidophilus* and Bifidobacterium to pregnant mice antagonized the detrimental effects of maternal serum IL‐1β, TNF‐α, and IL‐6 on embryonic intestinal development (Yu et al. 2020). The increased abundance of Bacteroidetes family S24‐7 in the intestines of pregnant female mice (E8.0) may enhance ASNS (Asparagine synthetase) gene expression, which encodes asparaginyl synthetase. This inhibition of L‐aspartic acid and phenylacetic acid accumulation promotes embryo implantation and placental development, thereby reducing risks of gestational diabetes, fetal growth retardation, and neurological disorders in maternal mice (Lin et al. 2024). Concurrently, Qianhong Ye's team demonstrated that enrichment of *Lactobacillus vaginalis* in sow intestines enhances uterine receptivity and benefits embryonic survival through the gut‐uterine axis (Ye et al. 2025). This conclusion is further supported by clinical studies: Ting Huang et al. found that elevated Actinobacillus abundance during pregnancy promotes maternal gut‐liver circulation via bile acids and arachidonic acid metabolites, benefiting embryonic neural development (Huang et al. 2024). However, maternal *Helicobacter pylori* infection during pregnancy may impair the absorption of folic acid and vitamin B12, as well as the production of short‐chain fatty acids, increasing the risk of neurological developmental disorders in offspring (Sun et al. 2024).

#### 2.4.4 Regulation of Fetal Development by Gut Microbiota

Normal gut microbiota during pregnancy can promote the production of regulatory T cells (Treg), maintain the maternal immune tolerance to the fetus, and promote the successful implantation of embryos and the subsequent development of the nervous system (Uchida et al. 2025; Xie et al. 2023; Liu, Li, et al. 2023; Nyangahu and Jaspan 2019; Li, Si, et al. 2025). In the fetal period, the gut microbiota imbalance caused by vancomycin can significantly reduce the number of maternal Treg cells and increase the risk of premature delivery. Butyrate supplementation can restore the number of Treg cells and reduce the rate of premature delivery (Wang, He, et al. 2017). Maternal gut microbiota can inhibit the over‐active IFN‐γ + and IL‐17+ T cell reactions at the maternal‐fetal interface by increasing the abundance of *Lactobacillus murinus* , a metabolite of tryptophan, and prevent fetal absorption caused by immune disorder (Brown et al. 2026).

#### 2.4.5 Regulation of Postnatal Development by Gut Microbiota

Maternal gut microbiota during pregnancy may also have an impact on neonatal intestinal development in the postpartum period. If antibiotics are used during pregnancy, it may inhibit the diversity of gut microbiota, which not only affects the health of mothers but also may affect the establishment of gut microbiota of newborns in the postpartum period (Hiltunen et al. 2022). In addition, early pregnancy exposure to antibiotics reduces *Lactobacillus reuteri* abundance in maternal intestines and affects offspring enteric nervous system (ENS) development through propionate‐mediated GPR41‐GDNF/RET/SOX10 signaling pathway, increasing susceptibility to colonic motility disorders, colonic epithelial damage, and water avoidance stress in adulthood. Supplementing *Lactobacillus reuteri* and propionate during pregnancy alleviates these symptoms (Zhang, Chen, et al. 2025). In certain pregnancy‐associated immune disorders such as maternal immune activation (MIA), supplementation with indole‐3‐propanoic acid (IPA) may reduce offspring susceptibility to colitis by increasing the abundance of probiotics including Bifidobacterium, Lactobacillus, and *Lactobacillus rhamnosus* (He, Ding, et al. 2025). Therefore, understanding the dynamic changes of maternal gut microbiota during pregnancy and their impact on maternal and infant health is a critical factor in developing effective interventions to improve maternal and child health outcomes.

While potential mechanisms like inflammatory responses, oocyte damage, and nutritional metabolism have been identified, the exact mechanisms linking maternal gut microbiota imbalance to embryonic abnormalities remain unclear. Elucidating these mechanisms holds profound implications for preventing fetal developmental disorders from an early life origin perspective.

## 3 The Role of Epigenetic Mechanisms in Maternal Gut Microbiota Influencing Prenatal Development

**FIGURE 3:**
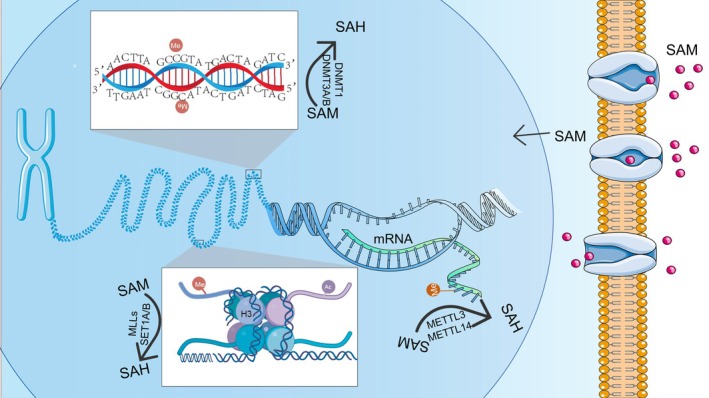
Illustrates SAM‐mediated epigenetic mechanisms during embryonic development. SAM regulates gene expression and transcription through DNA methyltransferases (DNMT1, DNMT3A/B), RNA methyltransferases (METTL3, METTL14), and histone lysine methyltransferases (MLLs, SET1A/B).

Numerous studies have demonstrated that diseases occurring during prenatal development are closely associated with epigenetic alterations (Table 3) (Lu et al. 2022; Zhang, Cao, et al. 2021; Qin et al. 2017; Ravaioli et al. 2022; Shi et al. 2021; Zhang, Liu, and Gao 2021; Hua et al. 2024; Lee et al. 2021; Chen et al. 2024). Epigenetics is the study of regulating gene expression through chemical markers—specifically DNA methylation, RNA methylation, histone modifications, and non‐coding RNA modifications—without altering DNA sequences, with these changes being inheritable to descendant cells. Research on maternal gut microbiota's impact on embryonic and fetal development has revealed that epigenetic regulation provides a novel perspective (Figure 3).

**TABLE 3:** Diseases occurring during embryonic development and their epigenetic mechanisms.

Disease	Cause of disease	Epigenetic mechanisms	Species	Document
Neural tube defect	Congenital structural defects caused by abnormal closure of neural tube in early embryonic development	The abnormal methylation of gDMR in the PEG10/SGCE cluster suppresses PEG10 gene expression and activates apoptosis pathways. The reduction of m6A RNA modifications disrupts the balance between cell proliferation and apoptosis by inhibiting the Wnt/β‐catenin signaling pathway. High methylation of microRNA‐124a promoter DNA suppresses its expression, thereby promoting apoptosis	Human Mouse Rat	(Lu et al. 2022) (Zhang, Cao, et al. 2021) (Qin et al. 2017)
21 Trisomy syndrome	Congenital chromosomal abnormalities caused by an extra chromosome 21	High methylation of CpG5 in the rDNA promoter reduces fetal cognitive development and survival rates. In fetal brain tissue, reduced m6A modifications on NRIP1 mRNA decrease its transcriptional degradation rate, resulting in abnormal elevation of NRIP1 expression	Human Human	(Ravaioli et al. 2022) (Shi et al. 2021)
Recurrent spontaneous abortion	Two or more consecutive spontaneous abortions	The reduced H3K27me3 at the RASA1 gene promoter leads to elevated RASA1 expression, which inhibits the Ras‐MAPK signaling pathway and consequently suppresses the proliferative and invasive capacities of trophoblast cells. The reduced EGR1 mRNA methylation results in decreased EGR1 protein expression, which leads to EPHB4 overexpression. This inhibits trophoblast cell proliferation, migration, and invasion while promoting apoptosis.	Human Mouse	(Zhang, Liu, and Gao 2021) (Hua et al. 2024)
Fetal growth restriction	The growth rate or weight of the fetus in the uterus is significantly lower than the normal level at the same gestational week	The hypermethylation of promoter DNA in the genes INS, MEG3, and ZFP36L2 suppresses gene expression, thereby affecting fetal development. The elevation of METTL3 enhances FOSL1 expression through m6A‐IGF2BP2‐dependent mechanisms, thereby impairing trophoblast invasion and migration.	Human Humans and mice	(Lee et al. 2021) (Chen et al. 2024)

### 3.1 DNA Methylation

DNA methylation refers to the chemical modification process where a methyl group is selectively added to specific sites of DNA molecules under the catalysis of DNA methyltransferases (DNMT). It primarily occurs at the 5 ‘‐C‐Phosphate‐G‐3’ (CPG) site and plays a significant role in gene regulation during prenatal development (Xu, Gong, et al. 2022; Monteagudo‐Sánchez et al. 2024). During dynamic changes in DNA methylation, the expression of specific genes is closely regulated. Different developmental stages and nutritional states may alter DNA methylation patterns, which may influence prenatal development through the placenta (Chen et al. 2022). For instance, maternal folic acid deficiency can impair the production of methyl donors like S‐adenosyl methionine (SAM), thereby affecting overall DNA methylation levels in placentas and embryos, hindering growth and development, and potentially leading to birth defects (Zhang, Liu, et al. 2025; Socha et al. 2024; van Otterdijk et al. 2023). Maternal choline intake increases the levels of stress‐related genes CRH and NR3C1DNA methylation and histone H3 lysine 9 dimethylation (H3K9me2) in placental and fetal tissues, thereby regulating fetal cortisol levels (Feng, Wu, et al. 2025; Jiang et al. 2012). Changes in DNA methylation not only impact gene expression in embryonic cells but may also influence the entire developmental process by affecting biological processes such as cell proliferation and differentiation (He, Ding, et al. 2025). For example, Sirt1 can counteract the expression of Dnmt3l to maintain balanced DNA methylation. When this balance is disrupted, it may result in excessive DNA methylation in embryonic stem cells, thereby inhibiting their differentiation capacity (Schulz et al. 2024). For instance, Sirt1 can antagonize the expression of Dnmt3l, maintaining DNA methylation in equilibrium. Once this balance is disrupted, it may lead to excessive DNA methylation in embryonic stem cells, thereby inhibiting their differentiation capacity (Heo et al. 2017). In studies exploring maternal effects on fetal development, the regulatory role of gut microbiota in DNA methylation has demonstrated significant research value (Li, Liu, and Liu 2024). Through multi‐omics analysis, oxidative stress (OS) genes associated with Crohn's disease (CD) are regulated by DNA methylation and host‐microbiota interactions (Xu et al. 2023). This suggests a potential link between DNA methylation and the host's gut microbiota in macro‐regulation, and elucidating their interaction may be key to understanding maternal‐fetal interactions. On one hand, maternal gut microbiota may influence embryonic DNA methylation patterns through SCFAs via their metabolites, thereby regulating the expression of embryonic development‐related genes (Zhang, Liu, et al. 2025). In Wenqian Guo's team's study, the accumulation of propionic acid in obese individuals' gut induced hypermethylation at the cg26345888 locus, which suppressed DAB1 gene expression and increased diabetes risk (Guo et al. 2022). Butyric acid can inhibit DNA methylation by downregulating DNA methyltransferase DNMT1 through phosphorylation of protein kinase M (PKM1) and extracellular signal‐regulated kinase (ERK) signaling (Mukhopadhya and Louis 2025). Maintaining stable maternal SCFAs during early pregnancy is crucial for normal embryonic neural development, as dysregulation may disrupt DNA methylation levels and affect neural development (Mukhopadhya and Louis 2025; Yang et al. 2020; Mirzaei et al. 2021). On the other hand, maternal gut microbiota may directly regulate embryonic and fetal DNA methylation processes (Zhang, Liu, et al. 2025). While most methyl donors can be produced through host‐endogenous pathways, certain probiotics (such as Bifidobacterium and Lactobacillus) can generate folic acid and other B vitamins (e.g., B2, B12), which serve as methyl donors for DNA methylation (Lapehn and Paquette 2022; Wu et al. 2023; Liu, Chen, et al. 2022). Consequently, changes in gut microbiota composition can influence the availability of SAM, thereby altering DNA methylation status in the host. For instance, in the absence of gut microbiota, DNA methylation levels in the gut significantly decrease. This hypomethylation is not due to reduced DNA methyltransferase activity, but rather results from decreased mononitrogenous metabolites originating from gut microbiota (Zhang, Liu, et al. 2025; Koren et al. 2024; Rosario et al. 2021). When germ‐free mice are re‐colonized with gut microbiota, DNA methylation in the gut is significantly enhanced (Koren et al. 2024; Stols‐Gonçalves et al. 2023). In summary, the potential interaction between maternal gut microbiota and DNA methylation provides new insights into the regulatory mechanisms of embryonic and fetal development. Future research in this field will not only help elucidate the role of epigenetics in developmental processes, but also lay the foundation for developing novel intervention strategies to improve maternal and offspring health.

### 3.2 RNA Methylation

RNA methylation refers to the chemical process of adding methyl groups to specific bases in RNA molecules (particularly messenger RNA‐mRNA). It primarily includes m6A, m5C, and m7G, which play crucial roles in RNA stability, translation efficiency, and alternative splicing, and are closely associated with the occurrence and development of various diseases such as cancer, Parkinson's disease, and epilepsy (Ling et al. 2025; Zhang, Wu, et al. 2025; Maqbool et al. 2025; Wang, Huang, et al. 2025; Wen et al. 2022). Recent studies have shown that m6A levels in vivo may be regulated by gut microbiota (Wang, Han, and Zhang 2025). The gut symbiotic bacterium *Enterobacter hormaechei* can regulate insulin receptor RNA m6A methylation through methionine (Zhang, Deng, et al. 2025). Lactobacillus and Bifidobacterium have also been shown to increase m6A levels in intestinal tissue RNA through folic acid (Wu et al. 2022). Conversely, changes in m6A modification enzyme activity can alter gut microbiota composition. For instance, knockout of demethyltransferase FTO increased the abundance of Lactobacillus, Porphyromonas, and *Helicobacter pylori* , reducing anxiety and depressive behaviors in mice (Sun et al. 2019). m6A modifications play a crucial role in early embryonic development and immune tolerance (Liu, Ge, et al. 2025; Liu, Zheng, and Liao 2022). Research by the team led by Boshi Feng demonstrated that knocking out the m6A reader protein YTHDF2 inhibits ROBO1 mRNA methylation, thereby affecting the differentiation of human embryonic stem cells (hESCs) into ectodermal cells (Feng, Chen, et al. 2025). In mouse studies, reduced m6A levels also suppress the Wnt/β‐catenin signaling pathway, leading to neural tube defects (Zhang, Cao, et al. 2021). The m6A regulatory network may also contribute to maintaining immune tolerance at the maternal‐fetal interface by modulating the functions of immune cells such as natural killer (dNK) cells, macrophages, and T cells in the decidua (Liu, Ge, et al. 2025). Regarding the role of maternal gut microbiota, studies have shown that compared to specific pathogen‐free (SPF) mice, mice with gut microbiota depletion exhibited m6A modification levels in brain tissue more similar to those of the embryonic state, characterized by significant upregulation of related enzymes (METTL3, METTL14, FTO, ALKBH5) (Wang et al. 2019). This suggests that maternal gut microbiota may participate in the dynamic regulation of fetal brain m6A modifications. Subsequent analyses in fetal mice (E18.5) further confirmed that maternal microbiota depletion affected the balance and overall m6A modification levels of m6A‐modifying enzymes in fetal brain and gut tissues (Xiao et al. 2022). Compared to DNA methylation, the maternal gut microbiota's regulation of prenatal RNA methylation remains at the hypothesis stage. Future research should clarify its critical regulatory window periods and sensitive targets, while exploring the feasibility of dietary or probiotic interventions during pregnancy. These efforts will enhance our understanding of early epigenetic warning mechanisms and intervention strategies, thereby preventing the onset of neurodevelopmental disorders and related conditions at their root causes.

### 3.3 Proteins and Non‐Coding RNAs Modification

**FIGURE 4:**
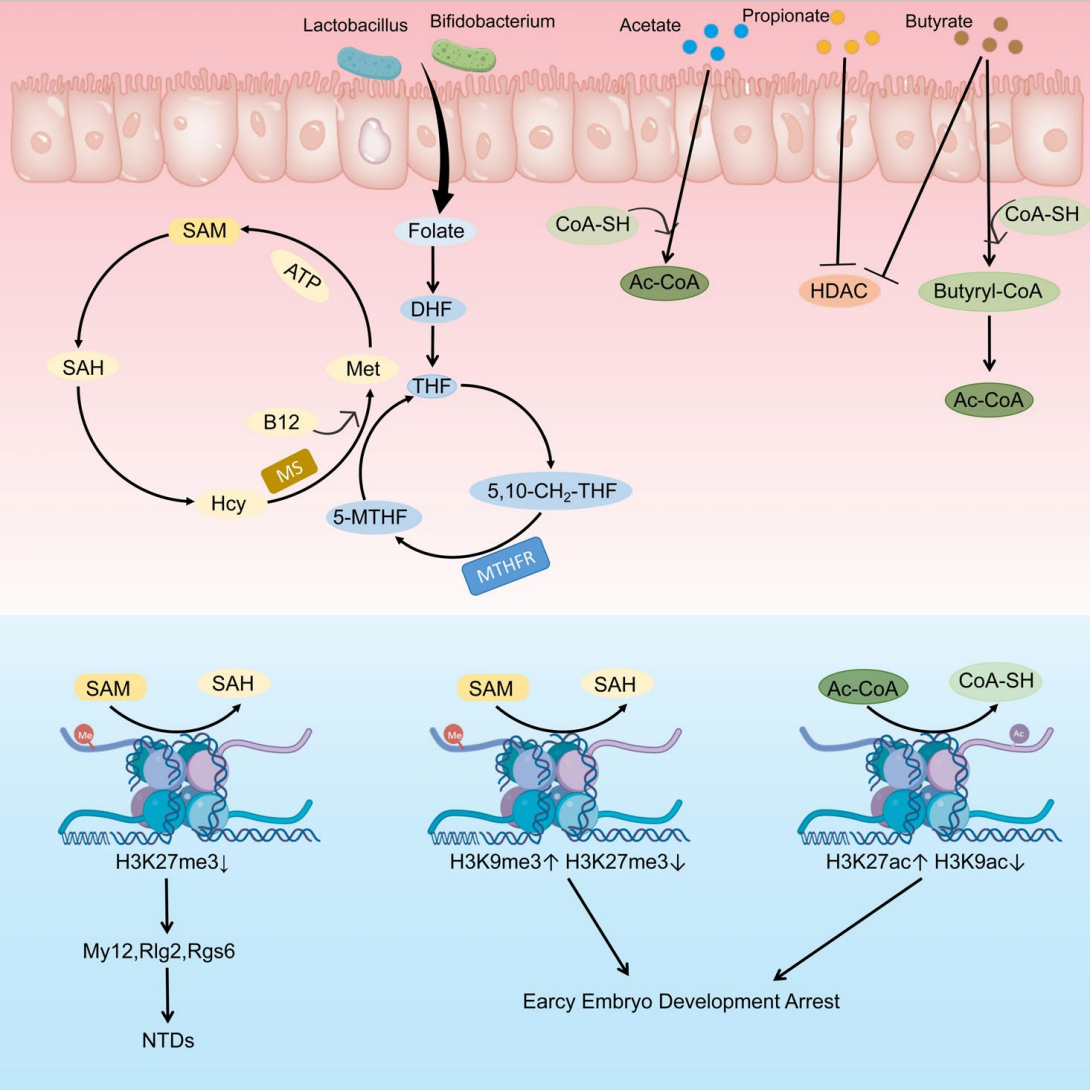
Illustrates the regulatory mechanisms of maternal gut microbiota and SCFAs on fetal histone modifications. Maternal gut bacteria, including Lactobacillus and Bifidobacterium, regulate embryonic histone methylation by modulating SAM (S‐adenosylmethionine), a key methyl donor. Meanwhile, SCFAs primarily control embryonic histone acetylation through two pathways: Regulating Ac‐CoA (acetyl‐CoA) production and modulating histone deacetylase (HDAC) expression.

Histone modifications refer to chemical alterations occurring on histone proteins (particularly the “tails” extending from DNA). These modifications do not alter the DNA sequence itself but precisely regulate gene “activation” and “inactivation” by adjusting DNA's coiling tightness (Ito et al. 2024; Zheng et al. 2023; Li, Yuan, et al. 2024; Xu et al. 2016). A fertilized egg must differentiate into over 200 distinct cell types (such as neurons, muscle cells, and skin cells), all containing identical DNA sequences. Histone modifications act as guides, controlling stem cell differentiation direction by regulating specific gene expression and silencing (Kubo et al. 2024; Wang et al. 2021; Wang, Xu, et al. 2017). For instance, H3K27me3 and H3K4me3 modifications play a central role in neural precursor cell differentiation. Increased H3K27me3 often suppresses expression of genes associated with neural tube closure (e.g., Myl2, Dlg2, Rgs6), leading to neural tube defects (Zhang, Liu, et al. 2025; Lin et al. 2023). During embryonic development, dynamic changes in histone modifications are crucial (Liu et al. 2018) (Figure 4). Research by Caiyun Wu's team demonstrated that early pregnancy exposure to chlorohexylpyridinium chloride (CPC) disrupts maternal‐to‐zygotic transition by increasing H3K9me3 and acH3K27 while reducing H3K27me3 and acH3K9 expression, ultimately causing developmental arrest in mouse preimplantation embryos (Wu et al. 2025). Similar to DNA methylation, histone modifications can also be regulated by gut microbiota and their metabolites. The gut microbiota tends to influence histone methylation levels, while intestinal metabolites more significantly regulate histone acetylation modifications. Similar to DNA methylation, gut microbiota regulates histone methylation by modulating the production of methyl donors such as SAM (Zhang, Liu, et al. 2025; Mirzaei et al. 2021; Wu et al. 2023). In histone acetylation, SCFA(e.g., butyric acid) produced by gut microbes can inhibit histone deacetylase expression, thereby promoting histone acetylation and facilitating cell proliferation and differentiation (Zhong et al. 2022; Nshanian et al. 2025). During pregnancy, alterations in maternal gut microbiota can also influence histone modifications in the fetus. For instance, reduced butyrate levels caused by changes in maternal gut microbiota may elevate H3K9ac and H3K27ac levels in fetal muscle tissue, thereby affecting the number of muscle fibers in fetal mice (Zuo et al. 2025). Consequently, by influencing histone modification states, the gut microbiota not only regulates gene expression but may also impact normal embryonic and fetal development through altered cell fate determination processes. Although the mechanisms by which gut microbiota influence histone modifications are currently well understood, direct and dynamic observational data on embryonic and fetal developmental mechanisms remain lacking.

Non‐coding RNAs (ncRNAs), including microRNAs (miRNAs), long non‐coding RNAs (lncRNAs), and small interfering RNAs (siRNAs), do not encode proteins but play crucial roles in regulating embryonic gene expression and cellular functions (Pan et al. 2025; Kaufmann et al. 2025; Li et al. 2022; Wang, Sun, et al. 2025). Recent studies have demonstrated that lncRNA and miRNA expression positively contribute to reducing hippocampal neuronal damage in mice and traumatic brain injury in humans (Wu et al. 2021; Shao et al. 2024; Zhang, Jiang, et al. 2024), suggesting ncRNAs may facilitate embryonic neural development. Their expression is regulated by DNA and RNA methylation (Xiong et al. 2021; Li et al. 2020). For instance, DNA methylation downregulates miRNA‐204‐5p expression, thereby inhibiting astrocytoma development (Jiang et al. 2021). Conversely, METTL3/YTHDF1‐mediated m6A modifications enhance lncRNA CHASERR and miR‐6893‐3p expression, promoting glioma progression (Wu, Fu, et al. 2024). Notably, gut microbiota and ncRNAs maintain close interactions (Wang, Qin, et al. 2024). Qiuke Hou's team discovered that the probiotic *Lactobacillus casei* subsp. paracasei LC01 suppresses intestinal epithelial cell permeability by downregulating miR‐144, thereby improving gut homeostasis (Hou et al. 2020). Interestingly, in Ting Li's team's study, downregulated miR‐30a‐5p could inhibit the growth of *Lactobacillus reuteri* in both mouse and human intestines (Li, Liu, et al. 2025). Additionally, long non‐coding RNAs (lncRNAs) are also regulated by gut microbiota (Li, Zhang, et al. 2025; Liu et al. 2019). For instance, Yuhao Wang's team demonstrated that compared to germ‐free mice, normal gut microbiota reprogram lipid metabolism by suppressing the expression of lncRNA Snhg9 in small intestinal epithelial cells (Quinn et al. 2023). This metabolic process (such as polyunsaturated fatty acids) is closely associated with embryonic nervous system development during pregnancy (Basak et al. 2022; Yu et al. 2021). Although various signs suggest that maternal gut microbiota may influence embryonic development through ncRNA regulation, the specific mechanisms remain unproven by robust animal experiments.

### 3.4 Plasticity of Epigenetic Regulation and Environmental Adaptation

Epigenetic plasticity refers to the dynamic regulation of gene expression through epigenetic modifications (such as DNA methylation, histone modifications, and non‐coding RNA regulation) without altering DNA sequences, enabling organisms to adapt to environmental changes or cell fate transitions (Yang and Wang 2021; Zhang, Fan, et al. 2025). During prenatal development, epigenetic modifications demonstrate remarkable plasticity, responding to maternal environmental changes and inducing lasting alterations in gene expression that influence health from early life through adulthood (Abdelnour et al. 2024; Bartman et al. 2025). As previously discussed, gut microbiota, acting as sentinels connecting humans with their environment, can promptly respond to environmental changes by modifying epigenetic modifications (Zhang, Liu, et al. 2025; Zhong et al. 2022; Cuevas‐Sierra et al. 2019). Moreover, epigenetic modifications not only function during embryonic development but may also exert intergenerational effects on offspring's phenotypes and adaptability (Xavier et al. 2019). This intergenerational inheritance of epigenetic traits allows offspring to retain adaptive memory in changing environments, thereby enhancing their survival and reproductive capabilities (Abdolmaleky et al. 2025). For instance, research by Yuta Takahashi's team demonstrated that DNA methylation targeting promoter‐associated CpG islands (CGIs) in mouse embryonic stem cells, along with the resulting phenotypic traits, can be maintained and transmitted across multiple generations (Takahashi et al. 2023). This indicates that epigenetic modifications in environmental adaptation are not merely transient responses but represent a long‐term adaptive process.

In summary, maternal gut microbiota can induce long‐term alterations in gene expression patterns by influencing epigenetic modifications in embryos and fetuses. These changes profoundly impact both early life and adult health. This remarkable plasticity not only provides rapid adaptation mechanisms to environmental changes but may also confer evolutionary advantages for species survival.

## 4 Epigenetic Mechanisms of Environmental Factors Affecting Maternal Gut Microbiota and Prenatal Development

### 4.1 Epigenetic Changes Caused by Environmental Exposure

In early embryonic development, drug exposure (such as morphine) may cause abnormal brain development and behavior in offspring by altering the function of the gamma‐aminobutyric acid (GABA) system (Zhang, Gu, et al. 2023; Wang, Jiang, et al. 2023). Cisplatin exposure, on the other hand, may damage the fetal auditory system through mitochondrial injury and reactive oxygen species (ROS) accumulation (Zheng et al. 2022). Recent studies increasingly demonstrate that environmental exposures may influence gene expression and regulation through epigenetic alterations (Liu, Gong, et al. 2024; Liu, Wang, et al. 2023; Li, Yan, et al. 2025; Zhang, Wang, et al. 2025). Environmental toxins primarily affect prenatal development‐related gene expression by modifying DNA methylation and histone modifications. For instance, methylmercury (MeHg) exposure during pregnancy can suppress the expression of neurodevelopment‐related genes (such as SYP and DLG4) through changes in DNA methylation and histone modifications, leading to embryonic neural developmental abnormalities (Kurita et al. 2024; Go et al. 2021). Environmental pollutants like heavy metals and pesticides have also been found to alter DNA methylation levels and affect the expression of genes related to development and metabolism, increasing the risk of birth defects and diseases (Zhang, Hu, et al. 2024; Nilsson et al. 2021; Li, Li, et al. 2025). Additionally, paternal environmental exposures before conception may influence offspring health through epigenetic marks in sperm, potentially predisposing them to metabolic disorders and immune system diseases in adulthood (Liao et al. 2025; Wu, Zhang, et al. 2024; Li, Gong, et al. 2025). For instance, paternal exposure to di(2‐ethylhexyl) phthalate (DEHP) before conception may alter methylation patterns in developmental gene families (such as Hox, Gata, Sox, etc.), thereby affecting the normal development of embryos (Oluwayiose et al. 2021). Similarly, paternal exposure to environmental toxins like polycyclic aromatic hydrocarbons (PAHs) and heavy metals can modify epigenetic marks to regulate gene expression associated with tumors and cell growth, increasing the risk of various diseases in offspring, including liver tumors, diabetes, and cardiovascular diseases (Nohara et al. 2020; Hong et al. 2024). In summary, epigenetic changes caused by environmental exposure not only significantly impact gene expression during embryonic and fetal development but may also exert long‐term effects on offspring health through paternal epigenetic inheritance. These studies provide new perspectives on the relationship between environmental factors and human diseases, while also offering crucial scientific evidence for future public health interventions.

### 4.2 Environmental Impact Transmission Mechanism Mediated by Maternal Gut Microbiota

Environmental changes can also indirectly affect prenatal development by altering the maternal gut microbiota composition (Mavel et al. 2025; Wang, Ma, et al. 2025; Wang, Zhang, et al. 2023). As a dynamic and sensitive ecosystem, the maternal gut microbiota serves as a crucial “environmental sensor” and “signal transducer” during pregnancy (Yan, Shi, et al. 2025). While not directly entering the embryo, it converts external environmental factors (such as diet, stress, and antibiotics) into internal biological signals (including metabolites and epigenetic markers), which are then transmitted to the fetus through the placenta, thereby influencing healthy embryonic development (Zhang, Chen, et al. 2025; Li, Lin, et al. 2024). For instance, exposure to prenatal antibiotics can increase the number of helper T cells 1 (Th1) while reducing the levels of helper T cells 2 (Th2), helper T cells 17 (Th17), and double‐positive T cells (FoxP3/RoRγT), leading to fetal and placental developmental abnormalities (Faas et al. 2023). Some studies have found that exposure to environmental toxins such as nicotine, bisphenol A, and LPS significantly alters the gut microbiota composition in pregnant mice, and this dysbiosis is associated with an increased risk of neonatal developmental defects (Zha et al. 2024; Zubcevic et al. 2022; Kar et al. 2022). Specifically, prenatal nicotine exposure increases Bifidobacterium abundance while reducing propionate production, thereby elevating the risk of miscarriage (Zubcevic et al. 2022). Exposure to bisphenol A may disrupt gut function and alter the gut microbiota composition, potentially impairing placental development and leading to compromised placental function and fetal growth (Zha et al. 2024). Simultaneous exposure to bisphenol A and graphene oxide (GO) may also disrupt the intestinal and placental barrier function, leading to increased embryo absorption rates and severe malformations (Zha et al. 2024; Liu, Wang, et al. 2024). Conversely, intake of folic acid, vitamins, and probiotics can mitigate the toxic effects on embryos to some extent (Cordero‐Varela et al. 2023; Huang et al. 2022).

In summary, maternal gut microbiota dysregulation caused by environmental exposure can influence prenatal development through multiple mechanisms, including immune system effects and potential epigenetic transmission to offspring. These findings provide new perspectives for studying maternal microbiome roles in embryogenesis, while also suggesting the importance of focusing on maternal health‐microbiome relationships in clinical practice to promote maternal and infant well‐being.

## 5 Maternal Nutrition and the Effects of Intestinal Microbiota on Epigenetic Regulation

### 5.1 The Regulatory Effect of Nutritional Status on Gut Microbiota During Pregnancy

Nutritional status during pregnancy significantly influences the composition and function of maternal gut microbiota, which in turn affects fetal health development (Ma, Chen, et al. 2025; Xu et al. 2025; Shi et al. 2025). Poor nutritional status, including both deficiencies and excesses, can lead to decreased microbial diversity and dysbiosis (Li, Zhang, et al. 2025). Specifically, pregnant women with overweight or obesity exhibit distinct gut microbiota differences compared to those with normal weight: their intestinal alpha diversity and abundance of beneficial bacteria like Firmicutes and Blautia are significantly reduced, while pro‐inflammatory groups (e.g., Desulfovibrio and Prevotella) show marked increases (Erlin et al. 2025; Zhou et al. 2023). These microbial alterations may disrupt nutrient transfer between mother and placenta, compromise maternal immune balance, and heighten risks of miscarriage and fetal developmental abnormalities (Yan, Li, et al. 2025; Cochrane et al. 2024). Nutritional deficiencies also impact maternal gut microbiota. Prenatal deficiencies in key nutrients such as vitamins and minerals may cause microbial imbalance, affecting fetal growth (Cui et al. 2024; Cheng et al. 2022). For instance, vitamin A deficiency increases Akkermansia abundance, elevating intestinal inflammation risks in offspring mice (Zhou et al. 2023). Conversely, folic acid deficiency reduces actinomycetes abundance in murine intestines, raising neural tube defect risks in embryos (Wang, He, et al. 2023). Furthermore, maternal nutritional status during pregnancy influences the production of gut microbiota metabolites. These metabolites cross the placenta into the fetal body, potentially having profound effects on fetal organ development (Zhu et al. 2025). For instance, vitamin D deficiency during pregnancy disrupts the mother's short‐chain fatty acid synthesis, increasing the risk of autism in offspring (Cui et al. 2024; Njunge and Walson 2023).

### 5.2 Nutrient‐Related Metabolites and Epigenetic Mechanisms

With the advancement of genome sequencing technology moving closer to personalized medicine, nutritional epigenetics has gained increasing recognition. This field investigates how dietary modifications alter gene expression patterns through nutrients and their metabolites influencing epigenetic mechanisms, thereby supporting healthy development and disease management (Sedley 2020). Vitamin B complex (including B6, B9, and B12) serves as a critical methyl donor for SAM synthesis, essential for maintaining DNA and histone methylation stability (Tanwar et al. 2020; Monasso et al. 2023). Insufficient intake of these nutrients may disrupt methylation patterns, elevating risks of metabolic disorders and cancer (Carlberg and Velleuer 2023; Ma et al. 2024). Nutrient availability also impacts embryonic development via placental barriers. For instance, glycine intake reduces H3K36me3 suppression in oocytes, promoting oocyte maturation and early embryonic development (Teng et al. 2024). Dietary fiber products similarly influence epigenetic regulation through their metabolites (Zhang, Zhang, et al. 2024). Butyrate, a key gut microbiota metabolite, enhances embryonic differentiation and muscle development by inhibiting histone deacetylase activity, thereby regulating histone acetylation (Yang et al. 2024). In conclusion, nutrition‐related metabolites form a complex relationship between maternal nutrition, gut microbiota and embryonic development by directly affecting DNA methylation and regulating histone modifications, providing a new perspective for understanding the relationship between maternal and fetal health.

### 5.3 Potential of Personalized Nutritional Interventions

**FIGURE 5:**
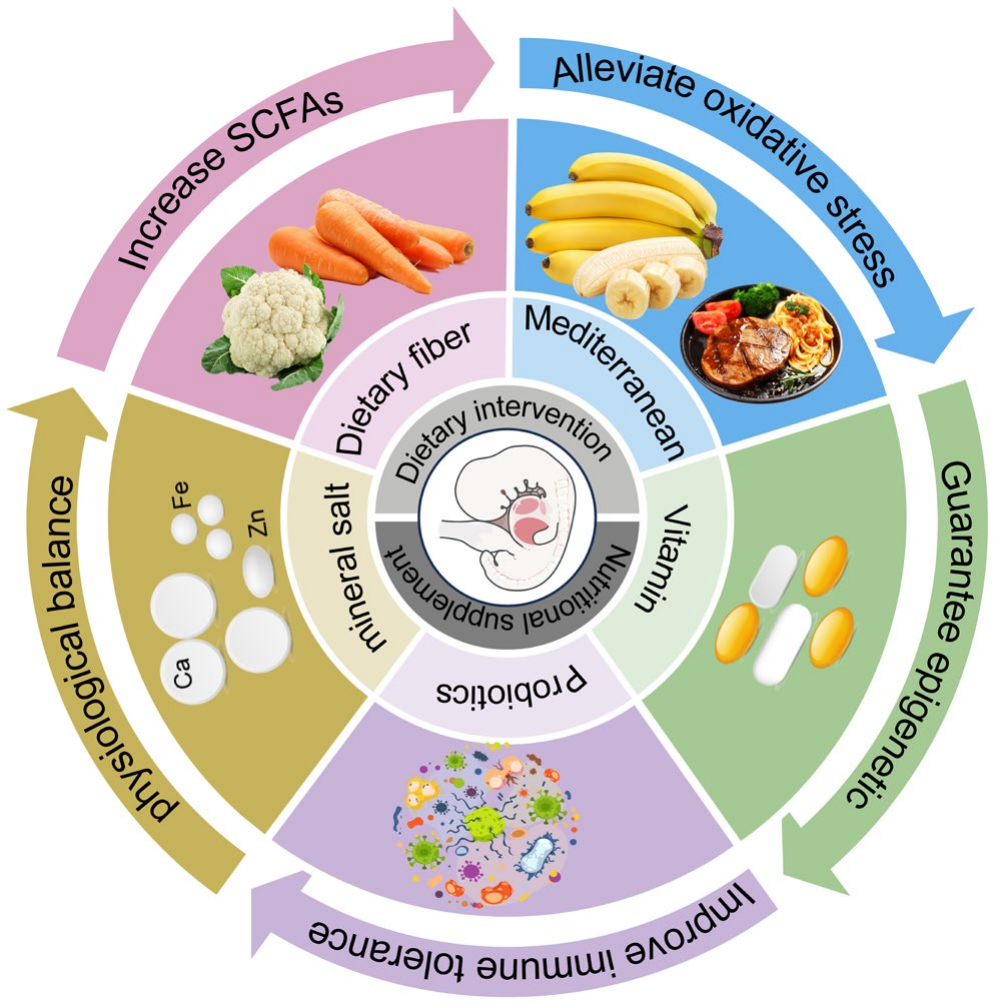
Nutrient supplementation and its mechanisms during pregnancy. During pregnancy, the mother improves the embryonic microenvironment through dietary fiber, Mediterranean foods, vitamins, probiotics, and minerals, thereby promoting embryonic growth.

The potential of personalized nutritional interventions to prevent pregnancy complications and fetal developmental defects during gestation has gained increasing attention (Luo et al. 2023). Maternal nutritional status during pregnancy not only affects maternal health but also significantly impacts fetal development and future health (Figure 5). Poor maternal nutrition may disrupt epigenetic modifications in fetuses, thereby increasing risks of chronic diseases such as cardiovascular disorders and diabetes (Coker et al. 2022; Wang, Liu, et al. 2025). Gut microbiota serves as the most sensitive indicator of nutritional status, with specific dietary patterns altering microbial diversity and metabolic functions, which subsequently influence host metabolic health (Wu, Cao, et al. 2024; Qu et al. 2023). For instance, the Mediterranean diet is believed to improve gut microbiota composition and may promote epigenetic “youthfulness,” alleviating oxidative stress and reducing chronic disease risks (Gensous et al. 2020). Nutritional interventions during pregnancy can modulate gut microbiota to influence epigenetic changes, thereby improving the embryonic developmental environment and lowering future non‐communicable disease incidence (Yang et al. 2025; Tzeng and Lee 2024). The key to personalized nutritional interventions lies in utilizing multi‐omics technologies to identify individual nutritional needs and their interactions with gut microbiota (He et al. 2024; Wang, Zhang, et al. 2024). By integrating genomic, transcriptomic, metabolomic, and microbiomic data, more targeted nutritional plans can be developed to optimize maternal nutrition during pregnancy and enhance maternal and infant health (He, Zhu, et al. 2025). This integrated approach not only reveals individual biological responses to nutritional interventions but also helps identify key health‐related biomarkers, thereby optimizing personalized nutrition plans (Yang et al. 2024). For instance, vitamin B12 and folic acid can alter the host's epigenetic modifications by influencing gut microbiota metabolic processes, thereby affecting embryonic nervous system development (Wang, He, et al. 2023; Rodríguez‐Cano et al. 2020).

In conclusion, maternal nutritional status during pregnancy plays a crucial role in regulating gut microbiota. Through appropriate nutritional interventions, we can enhance both the diversity and metabolic functions of maternal gut microbiota, thereby promoting healthy fetal development. Future research integrating multi‐omics data will explore how different nutritional interventions influence host epigenetic characteristics by modulating gut microbiota composition and function. Implementing personalized nutritional interventions could provide robust support for maternal and infant health, facilitating more precise health management and disease prevention strategies.

## 6 Challenges and Future Directions of the Impact of Gut Microbiota on Embryonic Development

### 6.1 Existing Challenges

Prenatal development is an exceptionally complex process. Due to ethical considerations in scientific research, current studies can only be conducted on mice. However, interspecies differences have always been a challenging factor to balance in scientific research. Although mice and humans share significant biological similarities, their gut microbiota fundamentally differ in composition and function (e.g., Bacteroidetes accounts for a lower proportion in mouse intestines compared to humans, while Proteobacteria makes up a higher proportion). Therefore, findings about probiotics, dietary interventions, or disease mechanisms derived from mouse experiments still require rigorous human validation before application to humans. Additionally, most epigenetic studies still lack key experiments (such as fecal microbiota transplantation) to establish causal relationships with gut microbiota.

### 6.2 Future Direction

The impact of gut microbiota on prenatal development can be analyzed through multi‐omics technologies to explore the relationship between maternal gut microbiota and epigenetics. Integrating metagenomics, methylomics, metabolomics, and transcriptomics helps reveal the regulatory networks between maternal microbiota and epigenetic processes. Metagenomics provides detailed information about microbial community composition, enabling analysis of bacterial species and abundance in the maternal gut to understand their effects on embryonic and fetal development (Shan et al. 2024; Luo et al. 2024). Methylomics offers insights into DNA, RNA, and histone methylation states during pregnancy, which are crucial for understanding gene expression and regulation in embryonic development (Minegishi et al. 2021; Urli and Greenberg 2025). Metabolomics transforms abstract microbial community data into measurable biochemical molecules, bridging epigenetic mechanisms with embryonic developmental outcomes (Zhang, Liu, et al. 2025; Yan, He, et al. 2025). Transcriptomics reveals dynamic changes in gene expression across developmental stages, helping identify precise mechanisms through which maternal microbiota influence embryogenesis (Chi et al. 2017; Gao et al. 2023). This multi‐omics integration not only provides a comprehensive perspective for studying maternal gut microbiota‐embryonic development interactions but also aids in understanding how environmental changes affect prenatal development. Future research should focus on mechanism elucidation, clinical validation, and optimization of intervention strategies to advance precision maternal and infant health management. In this process, interdisciplinary cooperation will be essential. Only by integrating the knowledge of nutrition, microbiology, molecular biology and other fields can we have a more comprehensive understanding of the role of maternal gut microbiota in embryonic development, and finally achieve individualized and precise health intervention programs.

## 7 Summary and Outlook

Prenatal development has long been a central focus in life sciences. Recent studies indicate that maternal gut microbiota, as a crucial microecological environment, may exert dynamic and precise regulatory effects on embryonic and fetal development. However, the molecular pathways through which maternal gut microbiota participate in this process during pregnancy remain a gap in current interdisciplinary research between reproductive biology and developmental biology. Building on nearly a decade of related studies, this paper systematically elucidates the regulatory mechanisms of maternal gut microbiota and its metabolites on embryonic and fetal development through epigenetic mechanisms, while further exploring how environmental and nutritional factors influence pregnancy outcomes via maternal gut microbiota. In the future, personalized nutrition‐mediated active reshaping of epigenetic trajectories in prenatal development may emerge as a novel strategy for preventing and treating prenatal diseases. To achieve this, current research requires deeper integration of multi‐omics technologies and in vitro/in vivo models to systematically reveal maternal‐fetal interaction networks, thereby providing a solid theoretical foundation for precision interventions in early life health.

## Funding

This work was supported by the Outstanding Youth Scientific Research Program for Universities in Anhui Province (2024AH020014), the High‐level Talent Scientific Research Startup Fundation of Wannan Medical University (12060201161), the Wannan Medical University Youth and Mid‐Career Research Funding (WK2023ZQNZ20), the Research Project of the Institute of Brain Science, Wannan Medical University (KF2024016), and the Scientific Research Fund for Undergraduate Students at Wannan Medical University (WK2024XS09).

## Conflicts of Interest

The authors declare no conflicts of interest.

## Data Availability

Data sharing not applicable to this article as no datasets were generated or analysed during the current study.
